# Structural Integrity of Particulate and Fiber-Reinforced Bulk-Fill Resin Composites

**DOI:** 10.3390/bioengineering13101109

**Published:** 2026-09-24

**Authors:** Abdulaziz Alshehri, Abdulrahman Alshabib, Yasser Alfawaz, Abdulaziz Alayed, Nick Silikas, Mohammed Alrabiah, Silvia Rojas-Rueda, Hamad Algamaiah

**Affiliations:** 1 Department of Restorative Dental Science, College of Dentistry, King Saud University, Riyadh 11545, Saudi Arabia; 443106465@student.ksu.edu.sa (A.A.); yalfaawaz@ksu.edu.sa (Y.A.); aaalayed@ksu.edu.sa (A.A.); 2 Biomaterials Science, Division of Dentistry, School of Medical Sciences, University of Manchester, Manchester M13 9PL, UK; nikolaos.silikas@manchester.ac.uk; 3 Department of Prosthetic Dental Science, College of Dentistry, King Saud University, Riyadh 11545, Saudi Arabia; mohalrabiah@ksu.edu.sa; 4 School of Dental Medicine, Ponce Health Sciences University, Ponce 00716, Puerto Rico; srojas@psm.edu

**Keywords:** bulk fill, fiber reinforcement, hygroscopic expansion, solubility, water sorption

## Abstract

Background: This study evaluated long-term water sorption, solubility, and hygroscopic expansion of bulk-fill resin composites (BRCs), with and without fiber reinforcement, at 37 °C, focusing on the influence of material composition on stability in aqueous environments. Methods: Five resin composites were tested: fiber-reinforced everX Flow (EVX); particle-reinforced SDR, Filtek Bulk-fill Flowable Restorative (FBF), and Filtek Bulk-fill Posterior Restorative (FBP); and conventional Filtek Z350 XT (FZ). Nine disk-shaped specimens per material were evaluated for water sorption and solubility according to ISO 4049. Hygroscopic dimensional changes were measured using laser scanning. Filler mass and degree of conversion were also assessed. Results: After 80 days, water sorption ranged from 13.79 to 30.13 μg/mm^3^. FZ showed the highest sorption and EVX the lowest. Flowable bulk-fill materials had lower sorption than packable materials. Solubility ranged from −3.10 to 0.73 μg/mm^3^. Hygroscopic expansion ranged from 0.64% to 1.51%, with EVX showing significantly lower expansion and FZ the highest. Conclusions: Short E-glass fiber-reinforced flowable composite showed lower hygroscopic expansion, yielding superior dimensional stability. Additionally, material solubility was highly matrix-dependent, with TEGDMA-free formulations demonstrating the lowest values. Conversely, applying the conventional composite beyond recommended increment depths severely impaired monomer conversion, which directly correlated with maximal water sorption and hygroscopic expansion.

## 1. Introduction

Modern dentistry has increasingly adopted resin-based composite materials (RBCs), driven by their favorable attributes, including superior esthetics and excellent physical and mechanical properties [1]. Since their introduction in the 1960s, RBCs have gained widespread acceptance, becoming the preferred option for direct restorations in both anterior and posterior dentition [2]. This expanded utilization of RBCs can be partially attributed to the 2013 Minamata Convention on Mercury, which mandates signatory parties to develop strategies for the phase-down of dental amalgam, thereby promoting the use of cost-effective and clinically efficacious mercury-free alternatives for dental restorations [3].

In recent years, bulk-fill composites (BRCs) have been developed to address the disadvantages of the conventional layering technique and facilitate restorative procedures. These materials permit placement in increments of 4–5 mm, which is particularly beneficial for the restoration of large cavities [4]. Enhanced light penetration through the material enables the polymerization of composite layers up to 4–5 mm in thickness without compromising the degree of conversion. This improved light transmittance is primarily achieved through strategies such as incorporating more reactive photoinitiators and augmenting material translucency [5]. Furthermore, this approach may also mitigate the formation of voids, which are prone to occurring at the interfaces between incrementally placed layers [6].

Resin composite materials face numerous challenges once placed in the oral cavity. These challenges include bacteria, temperature changes, food, saliva, and occlusal load can lead to accelerated degradation of the material, including biological, hydrolytic, enzymatic, and mechanical degradation processes, ultimately compromising their physical stability and mechanical properties [7]. While most resin composites are recognized for their chemical stability, chemicals present in the oral environment can be absorbed by the composite polymer networks. Subsequently, certain components from the resin matrix and filler particles may leach into the surrounding tissues. These phenomena are related to water sorption and solubility, which are considered fundamental factors that significantly influence the structural integrity of bulk-fill resin composites (BRCs) and, subsequently, their clinical performance within the oral cavity [8,9].

Water sorption in composite materials is a diffusion-controlled process, primarily occurring within the resin matrix [10]. Sorbed water within the polymer matrix can lead to hydrolytic degradation, potentially causing filler debonding or breakdown. This, in turn, compromises the material’s mechanical properties. Hydrolytic degradation involves the cleavage of chemical bonds within the resin or the plasticizing effect of water, which increases resin flexibility [11].

Two primary theories could explain the water sorption of RBCs: the Free Volume Theory, which posits that water molecules permeate into the existing micro voids within the polymer network. Their presence and interaction within these spaces can lead to a breakdown of the filler-matrix interface due to the plasticizing effect of water, compromising the material’s integrity [12]. The Interaction Theory (or Hydrogen Bonding Theory), on the other hand, suggests that water molecules chemically bond with the hydrophilic groups present within the resin matrix. This bonding results in hygroscopic expansion of the material and a corresponding increase in its mass [13]. Both mechanisms can contribute to the overall water sorption behavior of resin composites, influencing their dimensional stability, mechanical properties, and long-term performance in the oral environment [14].

A study investigating the water sorption of bisphenol A-glycidylmethacrylate (BisGMA), bisphenol A-ethoxylate dimethacrylate (BisEMA), and urethane dimethacrylate (UDMA) based monomers, with triethylene glycol dimethacrylate (TEGDMA) as a co-monomer, reported no statistically significant differences in water sorption values when conventional BisGMA and UDMA-based monomers were utilized, and BisEMA exhibited the lowest water sorption. Concomitant water solubility trends mirrored those observed for water sorption: BisEMA < UDMA < BisGMA [15]. Furthermore, it has been reported that BisGMA demonstrated higher water sorption compared to other monomers, while TEGDMA exhibited the highest solubility [16]. In a related investigation, when two brands of resin composite with the same filler content but differing monomer compositions were studied, the RBCs containing BisGMA and TEGDMA monomers displayed higher water sorption than the RBC formulated with UDMA and BisEMA [17,18].

Randolph et al. investigated the physical and mechanical properties of a diverse range of dental composites, focusing on filler content, filler shape, and filler distribution. Their investigation revealed that an increase in filler content correlated with a decrease in water sorption [19]. This finding was further corroborated by different studies that suggested that RBCs exhibited reduced water sorption as the filler load increased [20,21,22]. Furthermore, filler characteristics appear to play a significant role; it has been reported that unsilanated, prepolymerized fillers exhibited significantly higher water sorption values compared to other materials. Additionally, resin composites incorporating barium glasses and zirconia silicate fillers demonstrated greater susceptibility to aqueous attack than those containing quartz filler [23,24].

The recent advancement in bulk-fill resin composites (BRCs) includes the introduction of fiber-reinforced composites (FRCs), which enhance material properties through mechanisms such as crack deflection and fracture energy absorption [25]. Fibrous fillers are broadly categorized into micro-whiskers, short glass microfibers, ceramic nanofibers, polymer nanofibers, and nanotubes [26]. Notably, short glass fibers, when within the critical length range of 0.5–1.6 mm, impart both high translucency and superior mechanical properties compared to particulate-reinforced BRCs [27,28].

Studies investigating the water sorption of fiber-reinforced composites (FRCs) have yielded inconsistent findings [29,30,31,32]. While some reports indicate that FRCs exhibit lower water sorption compared to other bulk-fill materials [29,30], other investigations suggest a higher degree of water uptake in these materials [31,32]. Furthermore, differences in outcomes may be related to the specific material formulation, including resin-matrix chemistry, fiber and filler characteristics, curing conditions, specimen geometry, and water-storage duration. Although long-term water sorption and solubility have been evaluated in fiber-reinforced composites, studies simultaneously assessing these properties together with hygroscopic expansion in contemporary fiber-reinforced bulk-fill materials remain limited [23,31]. Therefore, the present objective was to determine the long-term water sorption and solubility, and the extent of hygroscopic expansion of bulk-fill resin composites, with and without fiber reinforcement, at 37 °C. The null hypotheses were as follows:

1. There are no differences in water sorption and solubility among the fiber-reinforced BRCs and other BRC composites after 80 days of water storage.
2. There are no differences in material expansion between the fiber-reinforced BRC composite and other BRC composites after 80 days of water storage.

## 2. Materials and Methods

### 2.1. Study Groups

This investigation employed five representative resin composite materials, detailed in Table 1 and outlined in the study design flowchart (Figure 1). Due to the limited market availability of fiber-reinforced resin composites, EverX Flow (GC Corporation, Tokyo, Japan) served as the exclusive representative of this material class. For a thorough comparative analysis, two established flowable bulk-fill materials, SDR (Dentsply Sirona, Charlotte, NC, USA), Filtek Bulk-Fill Flowable Restorative (3M ESPE, St. Paul, MN, USA) and one non-flowable bulk-fill material, Filtek One Bulk-Fill Posterior Restorative (3M ESPE, St. Paul, MN, USA) were included. A conventional nanofilled resin composite, Filtek Z350 XT (3M ESPE, St. Paul, MN, USA) was utilized as a negative control.

### 2.2. Tests for Standardization

To ensure standardization and thorough understanding of the materials investigated, the measurement of inorganic filler content and curing efficiency for each representative material was performed.

#### 2.2.1. Measurement of Filler Content

The ISO 1172:1996 and ASTM D5630-22 standards were employed to determine the mass percentage of inorganic fillers using the ashing technique for each group of resin composites (n = 2), as detailed in Table 1 [33,34]. Specimens with dimensions of 2 mm thick and 5 mm in diameter were fabricated using polytetrafluoroethylene (PTFE) molds. These molds were placed between two clear Mylar strip sections, which were sandwiched between glass slabs and compressed. The curing process was carried out using an Elipar S10 (3M ESPE, Seefeld, Germany) light-curing unit for 20 s on one side, delivering an irradiance of 1.2 W/cm^2^ (Bluephase meter, Ivoclar Vivadent, Schaan, Liechtenstein). Specimens were stored for 24 h in an incubator set to 37 °C. An electric furnace (Programat EP 5010, Ivoclar Vivadent, Schaan, Liechtenstein) was used to maintain a silica crucible at 630 °C for 30 min. The crucible was then cooled to room temperature (23 ± 2 °C) in a desiccator containing silica gel maintained at 37 ± 1 °C. The weight of the cooled crucible was measured using a precision digital balance (BM-252, A&D, Tokyo, Japan). Each composite specimen was then placed into the crucible, and the combined weight of the crucible and the specimen was recorded.

Subsequently, the crucible containing the specimen was subjected to a temperature of 630 °C for 30 min in the electric furnace to incinerate the organic matrix. After incineration, the crucible and its residual contents were cooled to room temperature in the desiccator before being reweighed. This process facilitates the calculation of the mass percentage of the inorganic filler by comparing the pre- and post-incineration weights. The inorganic filler content was calculated using Equation (1):

(1) $$Filler\;content\;(\%)=\frac{m3-m1}{m2-m1}\times 100$$

Equation (1). Inorganic filler content.

m1 is the initial mass, in grams, of the dry boat or crucible.
m2 is the initial mass, in grams, of the dry boat or crucible plus dried specimen.
m3 is the final mass, in grams, of the boat or crucible plus residue after calcination.

#### 2.2.2. Degree of Conversion

Three specimens of each resin composite presented in Table 1 were utilized (n = 3). Thus, a total of 30 disks (5 mm diameter and both 2.0 and 4.0 mm depths) were used for measuring the degree of conversion (DC) at different depths [35]. A split customized stainless-steel mold for DC samples was used for each group. The uncured materials were placed in the molds over the ATR (Bruker ALPHA-Platinum FT-IR Spectrometer, Bruker Optics GmbH, Ettlingen, Germany), a clear Mylar strip (Mylar Uni-strip, Dentsply Caulk, Milford, DE, USA), and a 1 mm thick glass slab was secured over the mold to flatten the surface and then gently pressed to remove excess material from the mold. The scans started for 20 s, then light cured from the top in contact with the mold surface for 20 s using Elipar S10 (3M ESPE, Seefeld, Germany). Thereafter, the sample was fixed in place over the ATR crystal to allow for a five-minute scan post-irradiation.

The absorbance and transmission peaks were obtained after five minutes using the reflectance mode of the FTIR spectrometer. The DC (%) was determined from the ratio of the absorbance intensities of the aliphatic C=C peak (1638 cm^−1^) against the internal reference aromatic C=C peak (1608 cm^−1^). The DC was calculated according to Equation (2) [36]:

(2) $$Degree\;of\;Conversion=(1-\frac{\left(1638\;{cm}^{-1}/1638\;{cm}^{-1}\;cured\right)}{\left(1638\;{cm}^{-1}/1638\;{cm}^{-1}\;uncured\right)})\times 100$$

Equation (2). Degree of conversion.

### 2.3. Water Sorption and Solubility

Water sorption (WSp) and solubility (WSl) evaluations were performed with modifications to the ISO 4049 standards due to the nature of the tested materials. Nine disk-shaped specimens were prepared for each material group via cylindrical molds (8.0 mm in diameter × 4.0 mm in thickness), which were set on a piece of clear Mylar strip film on a glass slab [21,37,38]. It was then covered with another piece of clear Mylar strip film while being pressed by another glass slab. Specimens were cured from the top for 20 s using an Elipar S10 (3M ESPE, Seefeld, Germany), with an irradiance of 1.2 W/cm^2^ delivering 24 J/cm^2^ radiant exposure. This single cure of the materials (within their mold) from the top was implemented for clinical simulation.

After photopolymerization, specimens’ weights (m1) were measured to an accuracy of 0.1 mg (Figure 2B). Samples were then placed into a desiccator with freshly dried white silica and kept at 37 °C for 22 h and at 23 °C for an additional 2 h (Figure 2C). Then, samples were weighed with an analytical balance to an accuracy of 0.1 mg. This cycle was repeated until each sample lost less than 0.1 mg of weight every 24 h. This constant mass in micrograms (mg), M1, was used as the initial mass of the specimen (Figure 2D). After 14 d, samples were completely dry; two measurements of the diameter, at right angles to each other, to an accuracy of 0.01 mm were recorded, and the mean diameter was calculated. Additionally, the thickness of the specimen to an accuracy of 0.01 mm at the center of the specimen and at four equally spaced points on the circumference was measured. Their average volume (V) was calculated in cubic millimeters (mm^3^) (Figure 2E). Each sample was then placed in distilled water (Figure 2G) and kept in an incubator at 37 °C until the materials reached the plateau (Figure 2H). The volume of water for immersion of the specimens was 10 mL per specimen. Specimens were then immersed in water for varying durations, and their mass were recorded at specific time points: 24 h, 48 h, 4 d, 7 d, 14 d, 21 d, 30 d, 70 d, 71 d, 73 d, 74 d, and 80 d. The immersion period was not pre-defined, specimen were kept in water until equilibrium (plateau) was attained, which was considered to have been reached when the change in mass of each specimen was less than 0.1 mg over any 24 h period. After the storage period, samples were removed, dried gently with absorbent paper, and weighed again to obtain M2 (Figure 2I). Subsequently, the samples were returned to the desiccator (Figure 2K) until a constant weight (M3) was achieved, a process that required 30 d, following the same protocol as for M1 (Figure 2L). Equation (3) is used to calculate water sorption:

(3) $$WSp=\frac{M2-M3}{V}$$

Equation (3). Water sorption (WSp).

Equation (4) is used to calculate water absorbed by a composite:

(4) $$WspC\%=\left(\frac{M2-M3}{M1}\right)\times 100$$

Equation (4). Water absorbed by the composite (WSpC%).

Equation (5) is used to calculate water absorbed by the polymer matrix:

(5) $$WSpM\%=\frac{WSp\%}{a}$$

Equation (5). Water absorbed by the polymer matrix (WSpM%).

Equation (6) is used to calculate water solubility:

(6) $$Wsl=\frac{M1-M3}{V}$$

Equation (6). Water solubility (WSl).

M1 = the mass of the specimen, in micrograms, prior to immersion in water; M2 = the mass of the specimen, in micrograms, after immersion in water; M3 = the mass of the reconditioned specimen, in micrograms; V = the volume of the specimen, in cubic millimeters; a represents the proportional weight of the polymer matrix in the composite.

**Figure 2:**
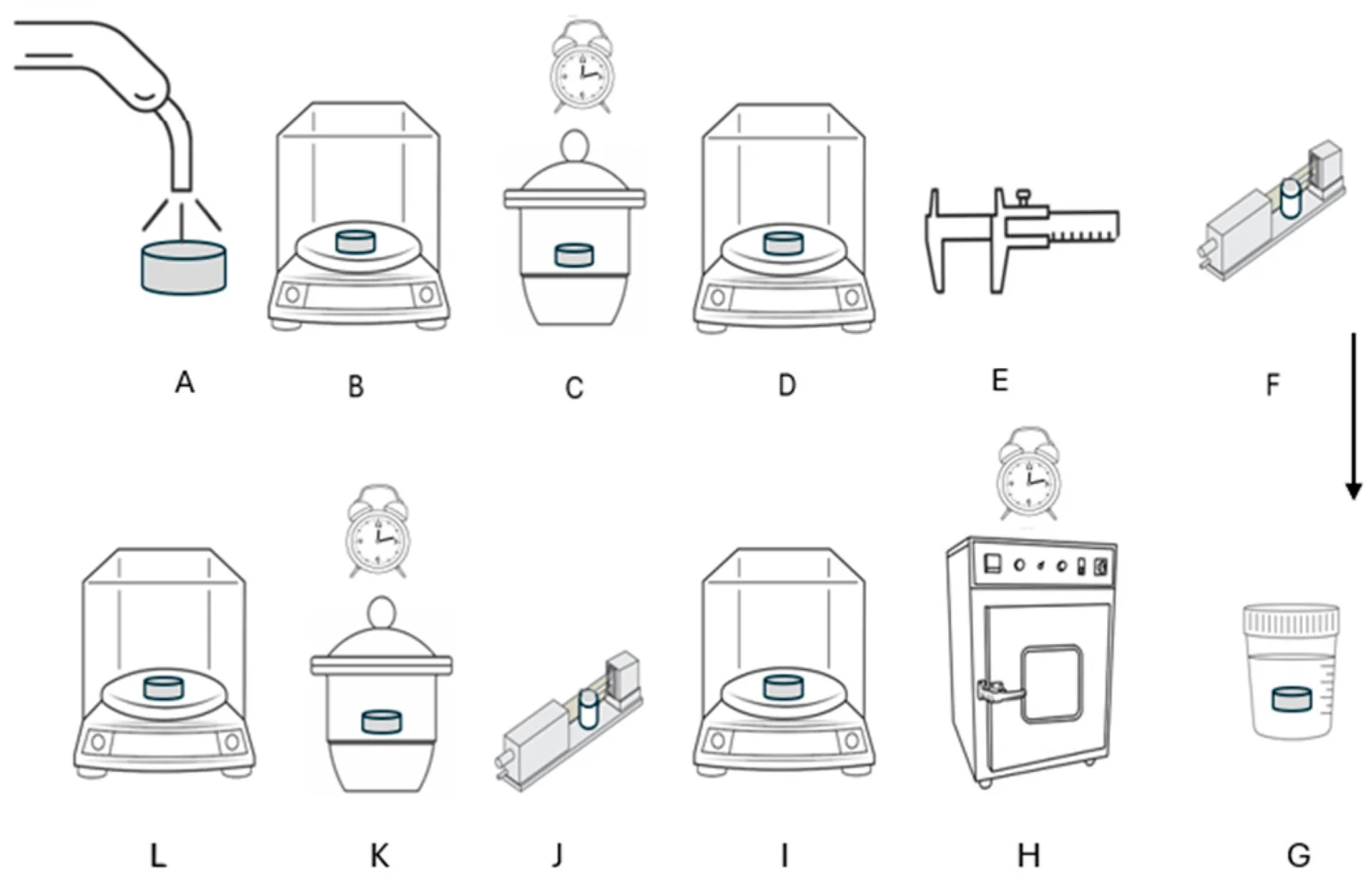
Schematic representation of the experimental sequence for the water sorption (WSp), solubility (WSl) and hygroscopic expansion measurements: (**A**) RC specimen. (**B**) Weights (m1). (**C**) Samples placed into a desiccator. (**D**) Initial mass (M1). (**E**) Average volume (V). (**F**) Laser scanning (d1). (**G**) Placed in distilled water. (**H**) Kept in an incubator (80 days). (**I**) Weighted after storage (M2). (**J**) Laser scanning (d2(t)). (**K**) Returned to the desiccator (30 days). (**L**) Weighted after desorption (M3).

### 2.4. Hygroscopic Expansion

Measurements of hygroscopic dimensional changes were conducted concurrently with water sorption tests using the laser scanning of a specimen disk (Figure 2) [39]. This technique entails rotating the specimen disk through a laser micrometer beam at multiple time intervals during the laser scanning process. A specially constructed non-contact laser micrometer was used to measure dimensional changes (Measuring Unit LSM-503s and Display Unit LSM-6200, Mitutoyo Corporation, Kawasaki, Japan). For every specimen, a total of 361 scans were recorded before the water sorption period, then the initial mean diameter (d1) was measured (Figure 2F). Specimens were then removed from the water, dried with filter paper, and then measured one minute after each time period. After recording the mean diameter (dt) at each time interval, the sample was returned to aqueous storage (Figure 2J). For every specimen, an average of 361 scans were recorded at every time interval. The percentage of diametral change was calculated using Equation (7).

(7) $$d(\%)=\frac{d_{\left(t\right)}-d_{1}}{d_{1}}\times 100$$

Equation (7). Percentage diametral change.

In this formula, d1 represents the mean diameter before water storage, and d(t) represents the mean diameter that is measured at each interval. The volumetric change was determined using Equation (8), which assumes isotropic expansion behavior:

(8) $$V\left(\%\right)=[(1+\frac{d\left(\%\right)}{100}{)}^{3}-1]\times 100$$

Equation (8). Percentage volumetric change.

In this formula, d (%) represents the percentage of diametral change.

### 2.5. Statistical Analysis

Sample size calculation was performed using G*Power v3.1.9.4 software (Düsseldorf, Germany). A power analysis revealed that a sample size of 9 per group for water sorption, solubility, and hygroscopic expansion met the constraints of f = 0.6, α = 0.05, and power = 0.85. Data are expressed as the mean and standard deviation, determined using SPSS Statistics software (version 22.0; IBM SPSS, Armonk, New York, NY, USA). The results were tested for normality and equality of variance using the Shapiro–Wilk and Levene’s tests to confirm the use of parametric tests. The results of water sorption, solubility, and hygroscopic expansion measurements of bulk-fill composites, with and without fiber reinforcement, were assessed and compared using one-way analysis of variance (ANOVA) followed by the Tukey post hoc test. All tests were performed at a significance level of *p* = 0.05. Pearson correlation coefficients were calculated to express the correlation between hygroscopic expansion and mass change for each material during 80 d water immersion.

## 3. Results

### 3.1. Tests for Standardization

The measurement of inorganic filler content and curing efficiency for each representative material is presented in Table 2.

### 3.2. Water Sorption

Figure 3 illustrates the percentage mass change exhibited by each resin composite material throughout the water sorption/desorption cycle. All composites demonstrated water uptake, resulting in a mass increase until equilibrium was reached at 80 d, confirming that equilibrium had been attained before the sorption period was ended for each tested material. Notably, with the exception of FZ, the reconditioned mass (m3) of all examined composites exceeded their initial mass (m1). At 80 d, water sorption values (presented in Table 3) ranged from 13.79 to 30.13 µg/mm^3^. Statistical analysis demonstrated that the highest water sorption was observed in the FZ negative control group (30.13 µg/mm^3^), followed by FBP (21.45 µg/mm^3^), FBF (17.38 µg/mm^3^), SDR (17.22 µg/mm^3^), and EVX (13.79 µg/mm^3^); the latter three bulk-fill flowable materials exhibited no significant difference (*p* > 0.05) (Figure 4).

### 3.3. Solubility

The solubility of the resin composites, presented in Table 3, ranged from −3.10 to 0.73 µg/mm^3^. Negative solubility values were recorded for all groups, with the exception of FZ, which exhibited a positive value (see Section 4 for discussion). Comparative analysis between the packable FBP material and the flowable materials (SDR, EVX, FBF) revealed that only SDR demonstrated the highest value, whereas EVX and FBF exhibited no differences compared to the FBP (*p* > 0.05). Overall, FBF (−3.10 µg/mm^3^) presented the lowest solubility, and FZ (0.73 µg/mm^3^) the highest (*p* < 0.05) (Figure 5).

### 3.4. Hygroscopic Expansion

The final hygroscopic expansion values, measured at 80 days, ranged from 0.64% to 1.51%. One-way ANOVA conducted on the data at 80 days of water immersion revealed that EVX exhibited a significantly lower hygroscopic expansion compared to all other materials (*p* < 0.05). Table 4 presents the mean and standard deviation for the volumetric hygroscopic expansion and mass change in all materials after 80 days of immersion at 37 °C. The percentage hygroscopic expansion for each material across the different time intervals is graphically depicted in Figure 6.

Intra-group comparison of the three flowable bulk-fill materials indicated that EVX (0.64%) demonstrated the lowest hygroscopic expansion, while SDR (1.22%) and FBF (1.15%) showed no statistically significant difference between their expansion values (*p* > 0.05). Furthermore, inter-group comparison of the flowable materials (SDR, EVX, FBF) with the control bulk-fill material FBP (1.00%) revealed no statistically significant difference in hygroscopic expansion (*p* > 0.05). While the hygroscopic expansion of the negative control group FZ was 1.51%, comparison with the flowable materials showed that only EVX presented significantly lower values, indicating no significant difference for the other flowable materials (*p* > 0.05).

## 4. Discussion

This study investigated the long-term water sorption, solubility, and hygroscopic expansion of various resin-based bulk-fill composites following an 80-day immersion period in distilled water at 37 °C. The materials evaluated were categorized into three main groups: a packable conventional composite (FZ), a packable bulk-fill composite (FP), and flowable bulk-fill composites. The flowable bulk-fill category was further subdivided to include fiber-reinforced material (EVX) and particulate bulk-fill materials (SDR, FBF).

The degree of conversion (DC), the mechanical characteristics of the material, and its biocompatibility, in addition to the physical performance of the RBC, will be affected by a reduced conversion rate, and reports of higher monomer elution have been documented [40]. Several studies have found that the higher the degree of conversion, the lower the number of unreacted monomers, and therefore, the lower the sorption and solubility [15,41,42]. In this study the DC of the investigated materials was assessed to ascertain its influence on long-term water immersion properties. Specimens were prepared at 2 mm and 4 mm depths, simulating common clinical depths. The results indicated a noticeable reduction in DC for the FZ material at the 4 mm depth, whereas other bulk-fill materials demonstrated comparable DC values across both depths. This finding was anticipated, as FZ is not indicated for bulk-fill application in 4 mm cavities, but rather for incremental placement [43]. Conversely, other BRCs exhibit enhanced light transmission, facilitating adequate polymerization up to 4–5 mm increments without compromising the degree of conversion [6]. Additionally, filler content was assessed in this study to provide further insight into the various factors that influence the properties of BRCs. Our findings demonstrated a 3–4% variation compared to the manufacturer’s report regarding the filler load of the investigated materials, which is in agreement with another study [31].

The water sorption values observed in the present study exhibited a negative correlation (r = −0.96) with the degree of conversion of the materials, as determined using the 4 mm mold. Specifically, FZ (30.13 μg/mm^3^) demonstrated the highest sorption value, followed by FBP (21.45 μg/mm^3^). Conversely, the flowable bulk-fill resin composites (BRCs), including SDR (17.22 μg/mm^3^), EVX (13.79 μg/mm^3^), and FBF (17.38 μg/mm^3^), exhibited comparably lower sorption values than the packable materials, with no significant differences observed among them (*p* > 0.05). As previously noted, the elevated sorption values observed for FZ were anticipated, given that this material was applied contrary to the manufacturer’s recommendations [43]. This outcome, despite FZ possessing the highest filler content among the tested materials, yet exhibiting the lowest DC, is consistent with the established literature, which indicates an inverse relationship between the degree of conversion and water sorption [15,42]. It is crucial to acknowledge, however, that the DC in this study was assessed at only five minutes post-irradiation. While it is well-documented that the degree of conversion typically progresses over time [44], water sorption can conversely affect the DC, particularly under conditions of substandard light curing or inadequate irradiance exposure, as may have occurred with this material during the water immersion [45]. It is worth noting that the specimens were kept dry at 37 °C for at least 14 days before immersion. This extended period was intended to allow post-cure polymerization and mass stabilization to occur before water exposure; thus, subsequent changes in mass and dimensions predominantly reflected water sorption, solubility, and hygroscopic expansion rather than early post-irradiation curing [38,44].

When comparing the water sorption behavior of flowable BRCs (SDR, EVX, FBF) with that of the packable BRC (FBP), the latter consistently exhibited higher sorption values. This occurred despite FBP containing a greater proportion of inorganic fillers and demonstrating a degree of conversion comparable to that of the flowable BRCs. Nevertheless, this finding agrees with other studies [46,47]. While the exact proprietary monomeric composition of FBP remains undisclosed by the manufacturer, it is reported to incorporate a long-chain aromatic urethane dimethacrylate (AUDMA) [48]. This inclusion is primarily intended to control the polymerization shrinkage by reducing the concentration of reactive functional groups within the resin system [49]. Nevertheless, this structural characteristic may inadvertently increase the free volume within the polymer network, thereby promoting water sorption [47,50]. Furthermore, FBP incorporates an Addition-Fragmentation Monomer (AFM) that forms a dynamic polymer network during polymerization, resulting in reduced shrinkage stress and enhancing mechanical strength [51,52]. AFMs introduce reversible covalent bonds that fragment and reform during polymerization, allowing the polymer to reorganize itself and relieve internal stresses, resulting in a more flexible and resilient material [53]. However, it was also noted that AFM-based polymers tend to exhibit increased water sorption within the resin matrix [53]. This observation could be attributed to the fact that increasing amounts of AFM slow down the reaction rate and reduce the final double bond conversion [54]. This interpretation is supported by the findings that when the effect of fillers was excluded by calculating the percentage of water absorbed by the polymer matrix alone (WSpM) rather than by the entire composite (WSpC), confirming that its polymeric matrix is strongly influenced by water storage. Furthermore, it is stated that the material incorporates non-agglomerated or non-aggregated silica fillers; however, the existing evidence suggests that nanofillers are inherently susceptible to aggregation due to their high surface area and energy, which can lead to silane agent hydrolysis upon exposure to water [55]. This hydrolytic degradation may lead to increased water absorption within the composite material [56].

The lower water sorption values observed for SDR material in this investigation are consistent with its documented higher degree of conversion (DC), as reported in previous studies [36,57,58,59]. The enhanced light transmission through SDR is attributed to its reduced filler content, which consequently diminishes light scattering within the material [60]. Furthermore, the specialized photo-active group embedded within the UDMA monomers of SDR significantly augments its light transmittance properties and facilitates greater energy delivery to the photoinitiators during the light cure process, contributing to its favorable DC and reduced water sorption [61,62].

FBF exhibited the lowest filler content among all investigated materials, concurrent with a lower degree of conversion (DC), a finding consistent with previous research [61]. Nevertheless, its water sorption was comparable to that of other flowable bulk-fill resin composites (BRCs). This outcome may be attributable to the resin matrix composition, specifically the inclusion of the Bis-EMA monomer, which is well-documented for its low water sorption characteristics [15,16,63]. Indeed, studies have indicated that materials containing a higher proportion of Bis-EMA exhibit reduced water sorption [64]. This attribute of Bis-EMA is due to the absence of hydroxyl (-OH) groups, leading to fewer and weaker hydrogen bonds with water molecules [65].

The EVX materials demonstrated the highest DC across all groups, and the highest filler load compared to the flowable BRCs SDR and FBF. This finding agrees with previous studies that reported higher DC with bulk filling materials incorporating fiber reinforcement [27,66]. This may be attributed to the presence of a relatively high percentage of E-glass fillers, which account for 25% of the total filler content, that contribute to great light transmission into the material [36].

In this study, the water solubility values of all BRC materials exhibited negative values. In contrast, FZ showed positive solubility values. The negative solubility values do not mean that the materials are insoluble; in fact, there are two possible explanations for these findings reported in the literature. The first posits that these values result from possible incomplete desiccation of the materials. This interpretation does not preclude the occurrence of minimal dissolution or eluate generation; rather, it suggests the low solubility [10]. The second explanation attributes negative solubility to a predominant water sorption capacity. That means when the magnitude of water uptake significantly exceeds the material’s inherent solubility, the absorbed fluid can effectively mask the true mass loss [23,67]. Furthermore, FBF (−3.10 μg/mm^3^) and FBP (−2.02 μg/mm^3^) exhibited the most favorable solubility values among the investigated materials. This observation is noteworthy as both materials reportedly lack TEGDMA in their resin matrix composition, unlike other materials in this study [68]. It is well documented that TEGDMA is a highly soluble monomer [16]. On the other hand, the positive solubility values of FZ (0.73 g/mm^3^) are consistent with the high water sorption found in this study. However, all materials demonstrated water sorption values below 40 μg/mm^3^, and their solubility did not exceed 7.5 μg/mm^3^ after the water storage period. Each composite material thus conformed to the recommended limits established by the ISO standard, despite undergoing an extended period of water immersion. Therefore, the first hypothesis that there are no differences in water sorption and solubility among the fiber-reinforced BRCs and other BRC composites after 80 days of water storage was partially rejected.

Despite the significance of hygroscopic expansion, this property has received limited research attention, and there is currently no standardized ISO test available [35]. A specialized technique, involving laser scanning of a specimen disk, has been developed for the measurement of hygroscopic expansion. This technique entails rotating the specimen disk through a laser micrometer beam at multiple time intervals during the laser scanning process [39].

Hygroscopic expansion occurs as water diffuses into the polymer network, leading to the separation of polymeric chains, particularly when water molecules can bond with the hydrophilic groups of the polymer [69]. It has been shown that water is absorbed by the resin phase, resulting in the volumetric expansion of the matrix to accommodate the absorbed fluid [31]. Moreover, hygroscopic expansion could compensate for the negative effects associated with shrinkage of the RCs by relieving the contraction stress, which is produced as a result of polymerization shrinkage and plays a significant role in bond failures for dental restorations [6]. The results of the present study found positive correlations between percentage mass and volumetric changes. The greatest volumetric change was observed in the FZ material (1.51%), whereas the lowest was noted in the EVX material (0.64%) across all other groups, correlating directly with their respective water sorption values. For that, the second hypothesis that there are no differences in material expansion between the fiber-reinforced BRC composite and other BRC composites after 80 days of water storage was rejected.

## 5. Conclusions

Within the limitations of this study, the following conclusions were drawn:

- Short E-glass fiber-reinforced flowable composite showed lower hygroscopic expansion, yielding superior dimensional stability. Additionally, material solubility was highly matrix-dependent, with TEGDMA-free formulations demonstrating the lowest values.
- Water sorption did not differ significantly among the flowable bulk-fill materials, unlike hygroscopic expansion.
- Applying the conventional composite beyond recommended increment depths severely impaired monomer conversion, which directly correlated with maximal water sorption and hygroscopic expansion.

## Figures and Tables

**Figure 1:**
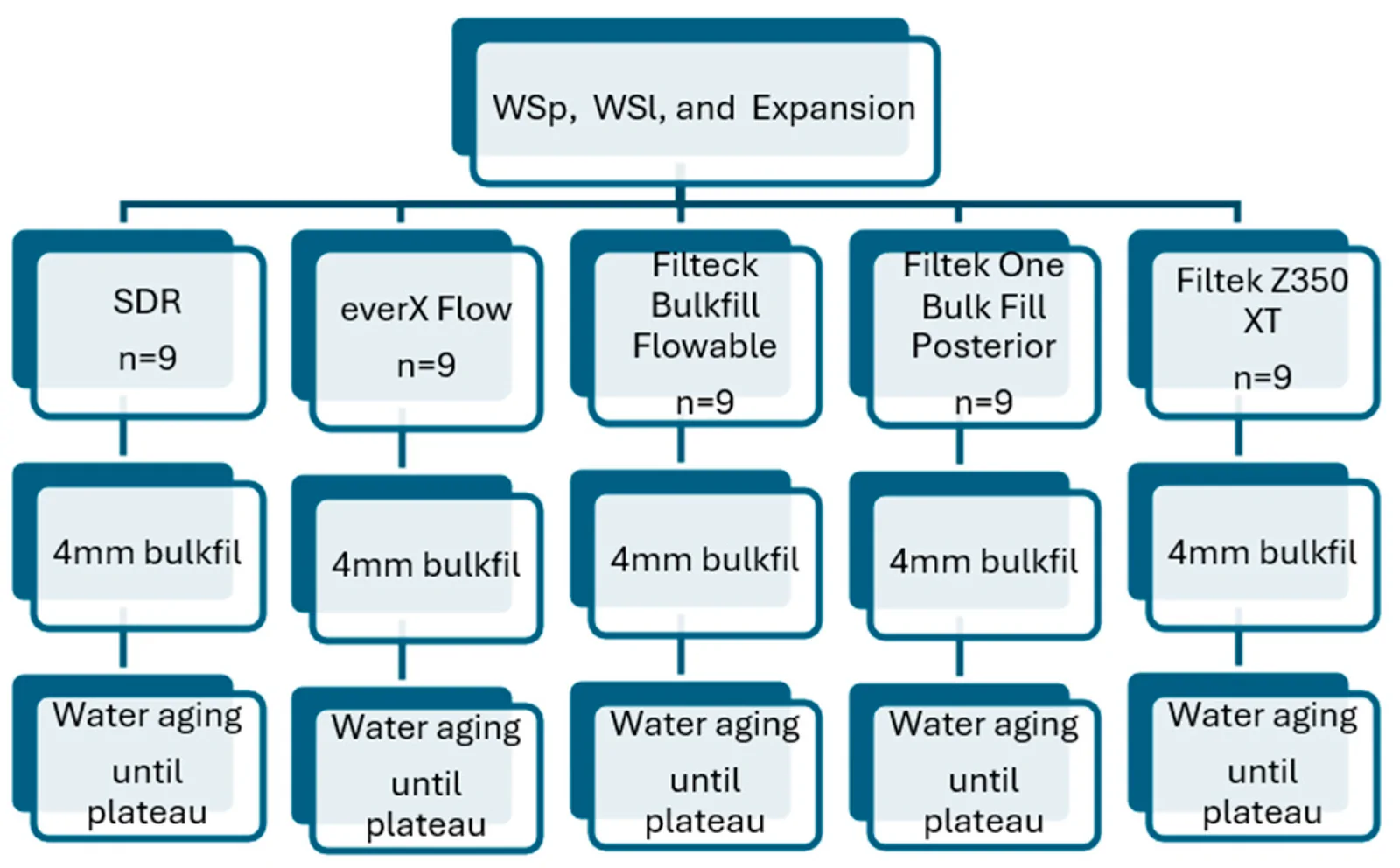
Flowchart of the study design (water sorption, solubility and expansion).

**Figure 3:**
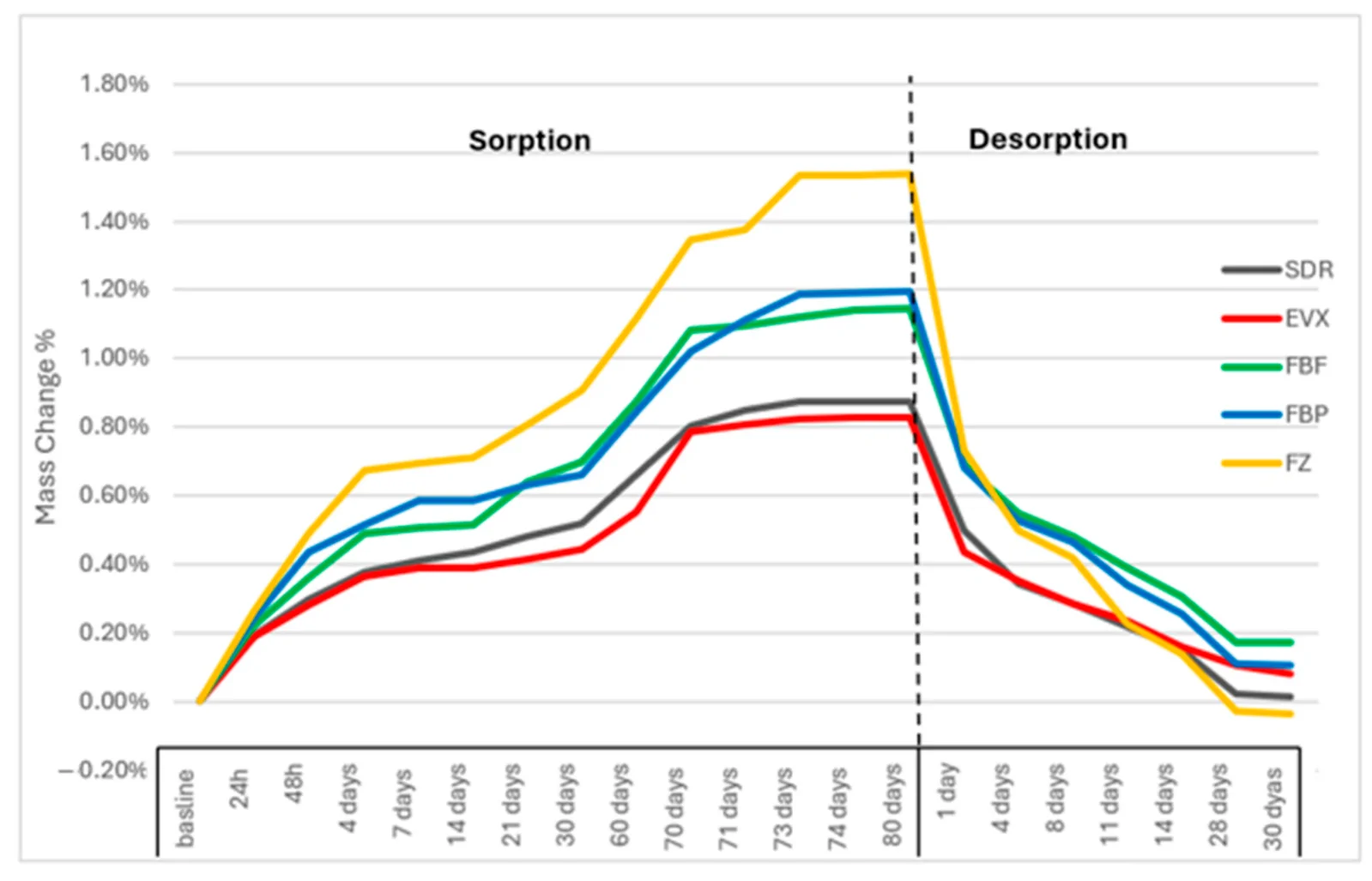
Mass change percentage with water sorption and desorption cycles.

**Figure 4:**
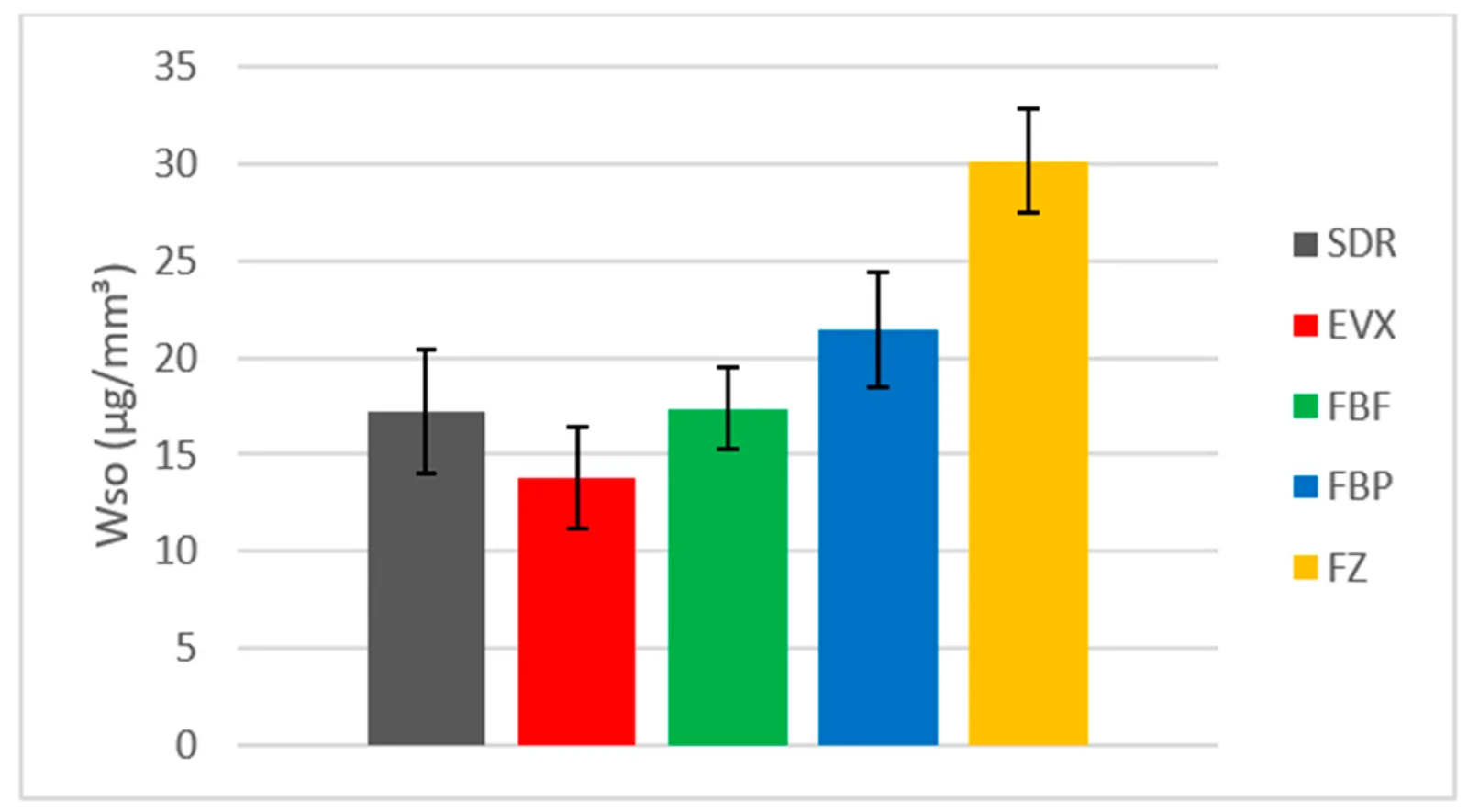
The mean values of water sorption(µg/mm^3^) for each material.

**Figure 5:**
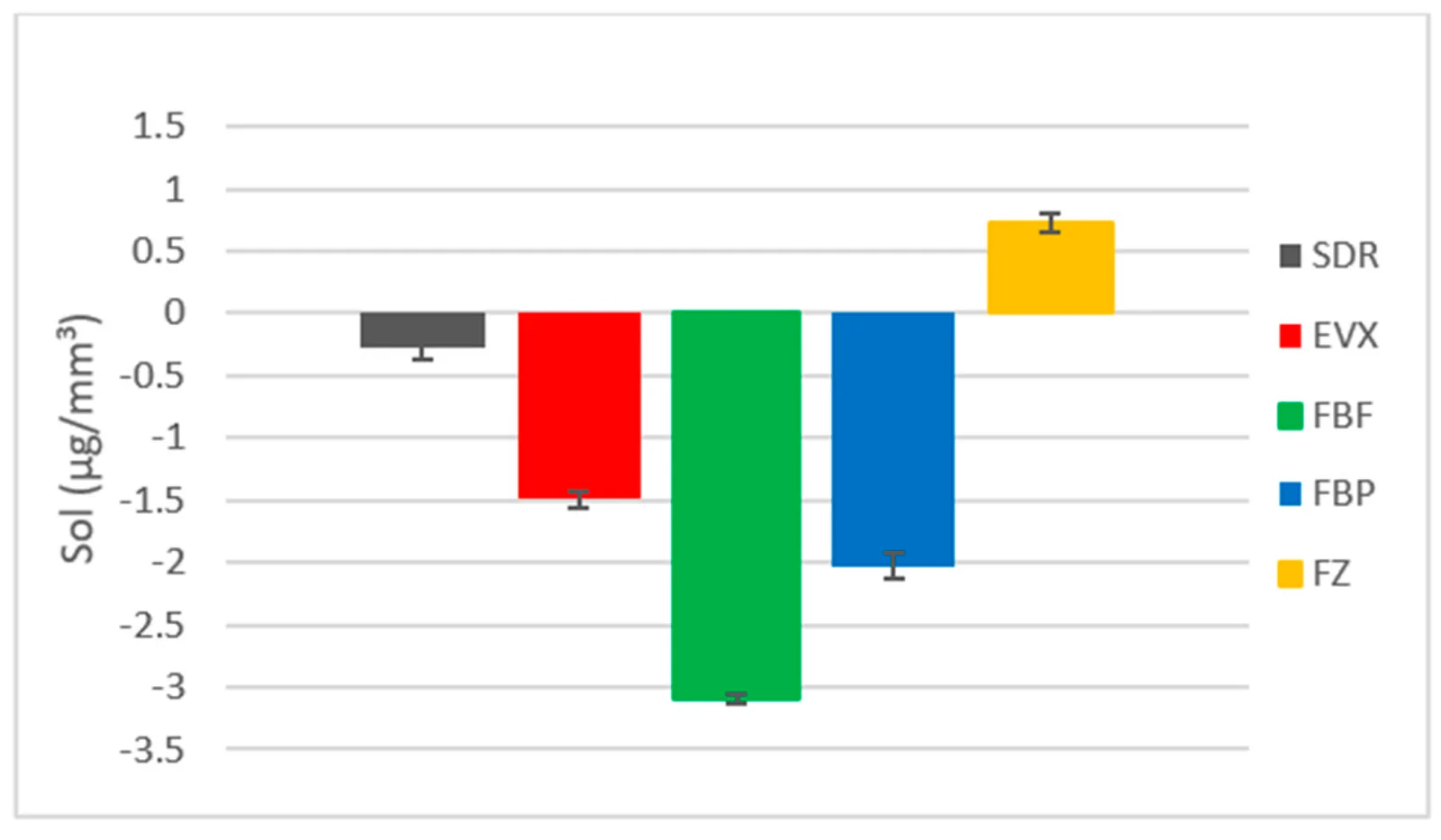
The mean values of water solubility (µg/mm^3^) for each material.

**Figure 6:**
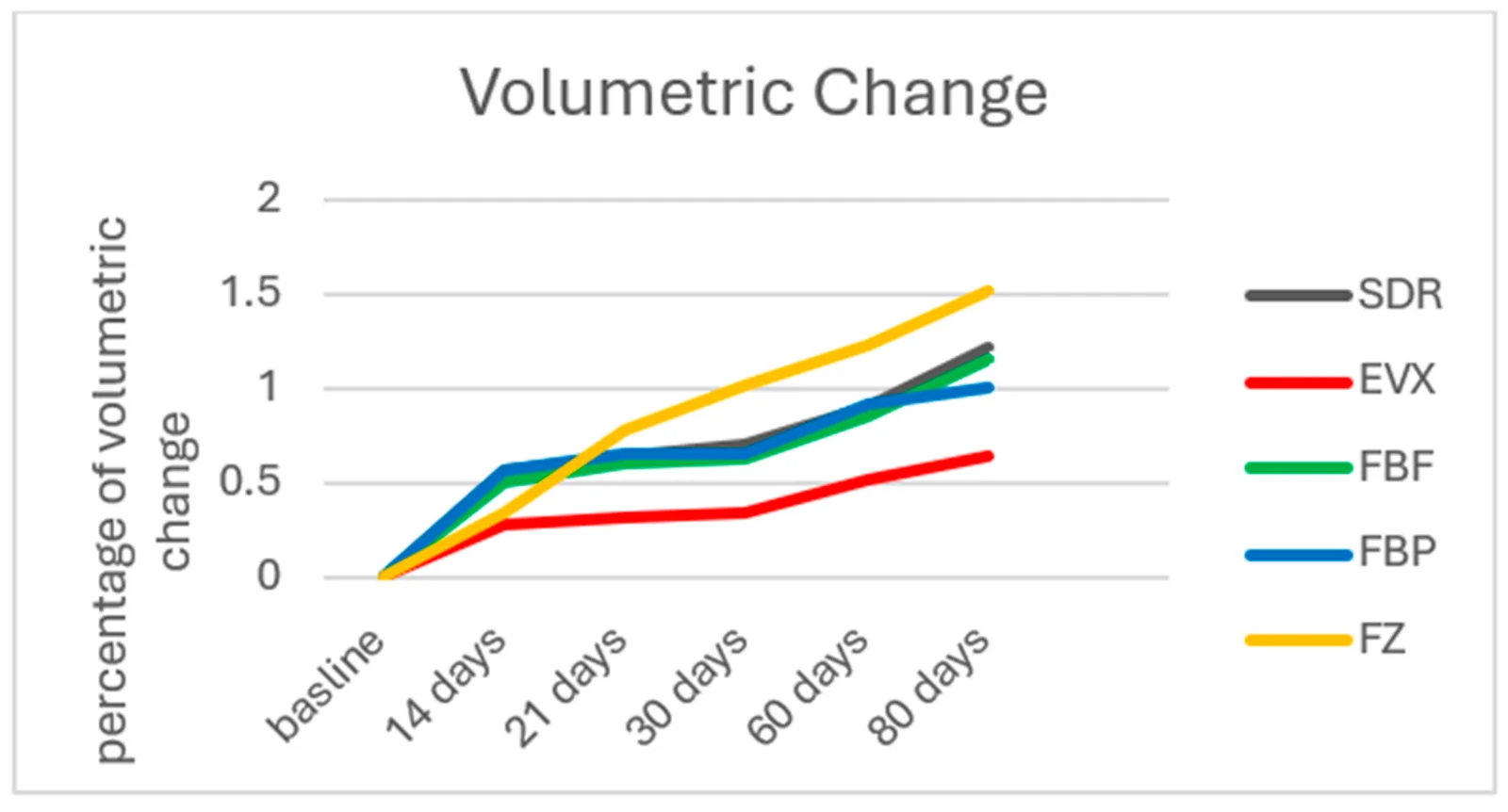
The volumetric change in all groups during the immersion interval.

**Table 1:** Detailed description of the materials.

Materials	Material Type and Code	Resin Matrix	Filler	Batch
SDR Dentsply	Flowable bulk-fill composite Code: SDR	Modified UDMA, dimethacrylate resin, difunctional diluents, EBPADMA, and TEGDMA	Filler content consists of barium and strontium alumino-fluoro-silicate glasses. Filler loading 68% by weight, 47.3% by volume. Inorganic filler particles range from 20 nm to 10 L	1501000007
everX Flow GC Corporation Tokyo, Japan	Flowable bulk-fill composite Code: EVX	Bis—MEPP, TEGDMA, UDMA	The fillers are a mix of short E-glass fibers and particle fillers, mostly barium glass, Silicon dioxide. The total filler rate of everX Flow is 70% in weight. (25% fibers 45% particles)	1912051
Filtek Bulk-Fill Flowable Restorative (3M ESPE)	Flowable bulk-fill composite Code: FBF	UDMA, BISGMA, Bis-EMA, Procrylat resin, 64.5% filler (0.01 to 5 µm—based on silica, zirconia and ytterbium trifluoride)	64.5 wt%/42.5 vol% zirconia, silica	N733627
Filtek Bulk-Fill Posterior Restorative (3M ESPE)	Packable nanofilled bulk-fill composite Code: FBP	ERGP-DMA, diurethaneDMA, and 1,12-dodecaneDMA	Filler content consists of nonagglomerated/nonaggregated 20 nm silica filler and 4 to 11 nm zirconia filler, aggregated zirconia/silica cluster filler, and ytterbium trifluoride filler agglomerate 100 nm particles. Filler loading 76.5% by weight, 58.4% by volume.	N682081
Filtek Z350 XT (3M ESPE)	Nanohybrid paste composite Code: FZ	Bis-GMA, UDMA, TEGDMA, PEGDMA, and Bis-EMA	Filler content consists of nonagglomerated/nonaggregated 20 nm silica filler and 4 to 11 nm zirconia filler and aggregated zirconia/silica cluster filler. Filler loading 78.5% by weight, 63.3% by volume.	N677462

**Table 2:** The mean and standard deviations of the filler weight percentage (wt. %) and the degree of conversion after five minutes scan.

Materials	Filler (wt. %)		Degree of Conversion %	
	Ashing Technique	Manufacturer	2 mm	4 mm
SDR	65.0 (0.5)	68.0	59.3 (0.3)	57.8 (0.2)
everX Flow	66.0 (0.3)	70.0	67.8 (0.6)	66.8 (0.1)
Filtek Bulk-Fill Flowable	61 (0.1)	64.5	57.8 (0.1)	55.6 (0.1)
Filtek Bulk-Fill Posterior	73.5 (0.4)	76.5	57.3 (0.6)	56.6 (0.2)
Z350	75.0 (0.3)	78.5	55.7 (0.1)	39.7 (0.3)

**Table 3:** Water sorption and solubility values.

Materials	WSp (µg/mm^3^)	WSL (µg/mm^3^)	WSpC%	WSpM%
SDR	17.22 (3.22) ^a^	−0.27 (0.08) ^c^	0.87 (0.13) ^a^	4.92 (0.44) ^a^
everX Flow	13.79 (2.61) ^a^	−1.48 (0.06) ^b^	0.75 (0.15) ^a^	4.06 (0.4) ^a^
Filtek Bulk-Fill Flowable	17.38 (2.11) ^a^	−3.10 (0.04) ^a^	0.98 (0.11) ^a^	4.46 (0.3) ^a^
Filtek Bulk-Fill Posterior	21.45 (2.99) ^b^	−2.02 (0.1) ^a b^	1.09 (0.16) ^a^	8.09 (0.5) ^b^
Z350	30.13 (2.66) ^c^	0.73 (0.07) ^c^	1.58 (0.14) ^b^	12.05 (0.57) ^c^

The same superscript small letters indicate a homogeneous subset (Columns) (*p* > 0.05).

**Table 4:** The mean values (SD) of the mass change and the volumetric change in resin composite groups after 80 days of storage in distilled water at 37 °C.

Materials	Mass Change %	Volumetric Change %	Pearson Correlation Coefficient
SDR	0.88 (0.01)	1.22 (0.32) ^b c^	0.43
everX Flow	0.83 (0.01)	0.64 (0.17) ^a^	0.46
Filtek Bulk-Fill Flowable	1.14 (0.01)	1.15 (0.33) ^b c^	0.44
Filtek Bulk-Fill Posterior	1.19 (0.01)	1.00 (0.15) ^a b^	0.38
Z350	1.54 (0.01)	1.51 (0.28) ^c^	0.63

The same superscript small letters indicate a homogeneous subset (columns) (*p* > 0.05).

## Data Availability

The data presented in this study are available on request from the corresponding authors.
